# Ixekizumab as a successful treatment in pediatric generalized pustular psoriasis

**DOI:** 10.1186/s13052-024-01835-2

**Published:** 2025-02-11

**Authors:** Maria Esposito, Paolo Antonetti, Emanuele Vagnozzi, Andrea De Berardinis, Roberta Bertelli, Francesco Brancati, Maria Concetta Fargnoli

**Affiliations:** 1 Dermatology, Department of Biotechnological and Applied Clinical Sciences, University of L’ Aquila, Via Vetoio, Coppito 2, L’ Aquila, 67100 Italy; 2 General and Oncologic Dermatology San Salvatore Hospital, ASL 1 Abruzzo, L’ Aquila, Italy; 3 Laboratory of Human Genetics, IRCCS Giannina Gaslini, Genoa, Italy; 4 Human Genetics, Department of Life, Human Genetics, Health and Environmental Sciences, University of L’ Aquila, L’ Aquila, Italy; 5 IRCCS San Raffaele, Roma, Italy

**Keywords:** Generalized pustular psoriasis, IL-17A, Ixekizumab, Biological therapy, Pediatric psoriasis, Case report

## Abstract

**Background:**

Generalized Pustular Psoriasis (GPP) is an autoinflammatory, multisystemic disease, characterized by widespread eruption of neutrophilic pustules on erythematous base, accompanied by systemic symptoms such as fever, leukocytosis, arthralgia, and general malaise. Globally, the disease is rare, particularly in children. If not adequately diagnosed and treated, systemic inflammation and multiorgan involvement can be life-threatening. The pathogenesis of GPP mainly involves the innate immune system, with inflammatory processes and neutrophil activation driven primarily by IL-36, but also by IL-1, TNF-alpha, IL-17 A. In particular, IL-17 A acts as a potent inducer of neutrophil recruitment. We report the case of a 7-years-old girl with GPP successfully treated with Ixekizumab, an IL-17 A antagonist.

**Case presentation:**

A 7-years-old girl with an history of plaque psoriasis came to our attention for the sudden appearance of erythematous patches surmounted by pustules on the trunk and lower limbs, following repeated episodes of purulent tonsillitis. We started therapy with cyclosporine at a dosage of 3,5 mg/kg/day, with no clinical benefit and progression of manifestations to a sub-erythrodermic state after 2 weeks. Blood tests showed neutrophilic leukocytosis, and the patient experienced hyperpyrexia and malaise. Since ixekizumab was recently approved for pediatric use in patients with moderate to severe plaque psoriasis, we started therapy with 80 mg Ixekizumab, combined with prednisone at a dosage of 12.5 mg/day, gradually tapered until discontinuation after 15 days. A second dose of Ixekizumab 40 mg was administered at week-4, according to the indication of ixekizumab in pediatric plaque psoriasis. At week-8 the patient achieved complete remission of skin manifestations and normalization of blood count. After achieving a stable remission, at week 36 we decided to increase the administration interval to 6 weeks. The patient is still on therapy with ixekizumab 40 mg every 6 weeks, maintaining complete remission during a 52-week follow-up, without safety concerns.

**Conclusions:**

This report supports the use of ixekizumab as a safe and effective option, both in the short and long-term, in the treatment of GPP, even at pediatric age. Larger studies are needed to confirm this positive, real-life experience.

**Supplementary Information:**

The online version contains supplementary material available at 10.1186/s13052-024-01835-2.

## Background

Generalized Pustular Psoriasis (GPP) is an autoinflammatory, multisystemic disease, characterized by a diffuse eruption of neutrophilic pustules on an erythematous base on non-acral skin, accompanied by systemic symptoms [1]. Globally, the disease is rare, especially in children [1]. If not properly diagnosed and treated, systemic inflammation and multiorgan involvement in GPP can be life-threatening. Potential triggers for flares include drugs use or discontinuation, infections, stress and pregnancy (2–3). Although the exact pathogenesis is unclear, it is known that GPP mainly involves the innate immune system, with autoinflammatory processes and neutrophil activation driven by interleukin (IL)-1, TNF-alpha, IL-17 A and, above all, IL-36^3–4^. Notably, in a subset of patients with phenotypes overlapping GPP, genetic variants in the *IL36RN* gene (encoding IL-36 receptor antagonist, also known as DITRA), *CARD14* (encoding Caspase Recruitment Domain-containing protein 14) and *AP1S3* (encoding a subunit of the adaptor protein complex 1 promoting vesicular trafficking between the trans-Golgi network and the endosomes) have been reported, outlining genetic heterogeneity of the condition, in addition to an autoinflammatory pathogenesis [1–4]. Ixekizumab is an IL-17 A antagonist recently approved for pediatric use in patients with moderate to severe plaque psoriasis [5]. We report the case of a 7-years-old patient with GPP successfully treated with ixekizumab.

## Case presentation

**Fig. 1:**
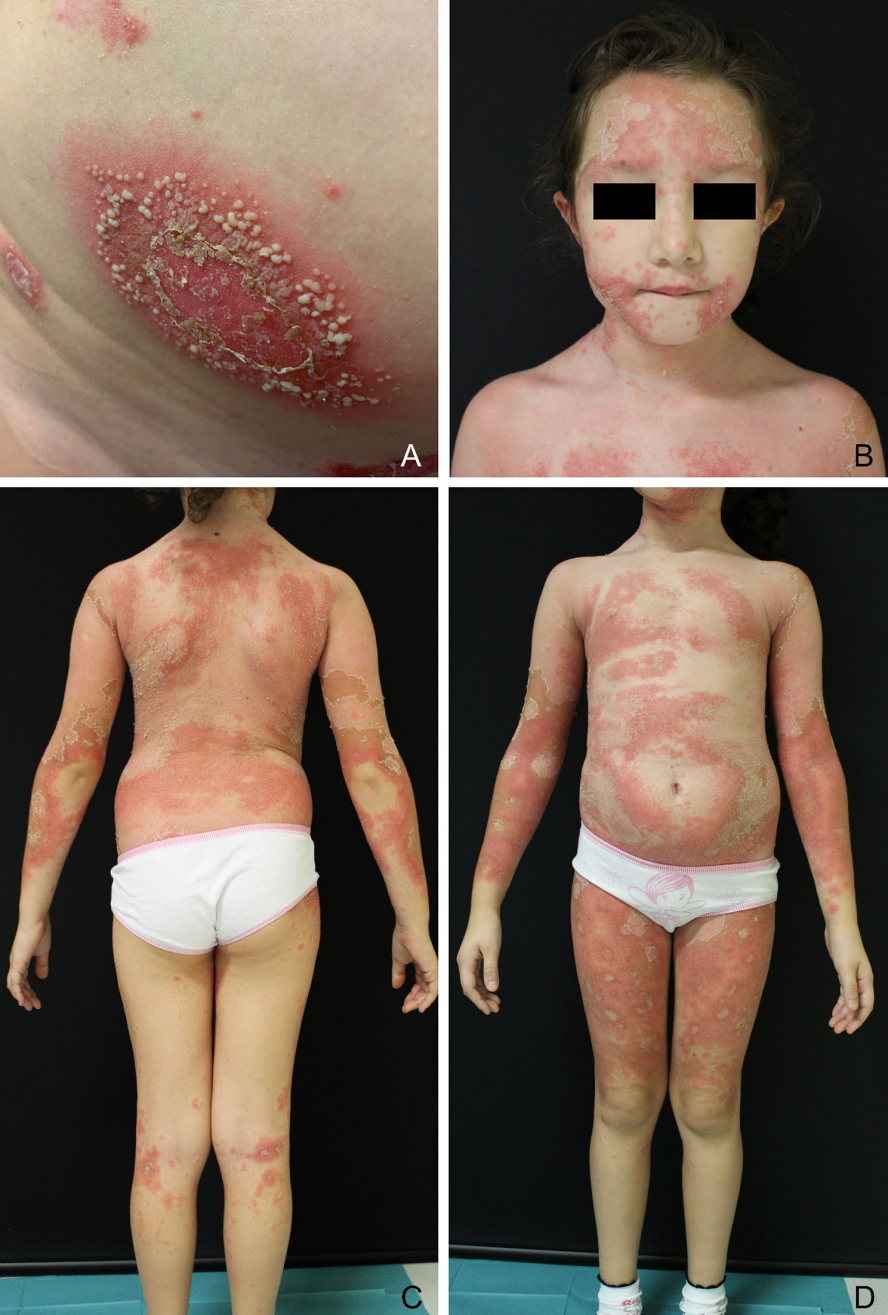
Onset manifestations of generalized pustular psoriasis. Few erythematous patches surmounted by pustules on the trunk and lower limbs (**A**). Severe and diffuse sub-erythrodermic state with residual pustules and desquamation after 2 weeks from onset with coexist (**B**-**D**)

**Fig. 2:**
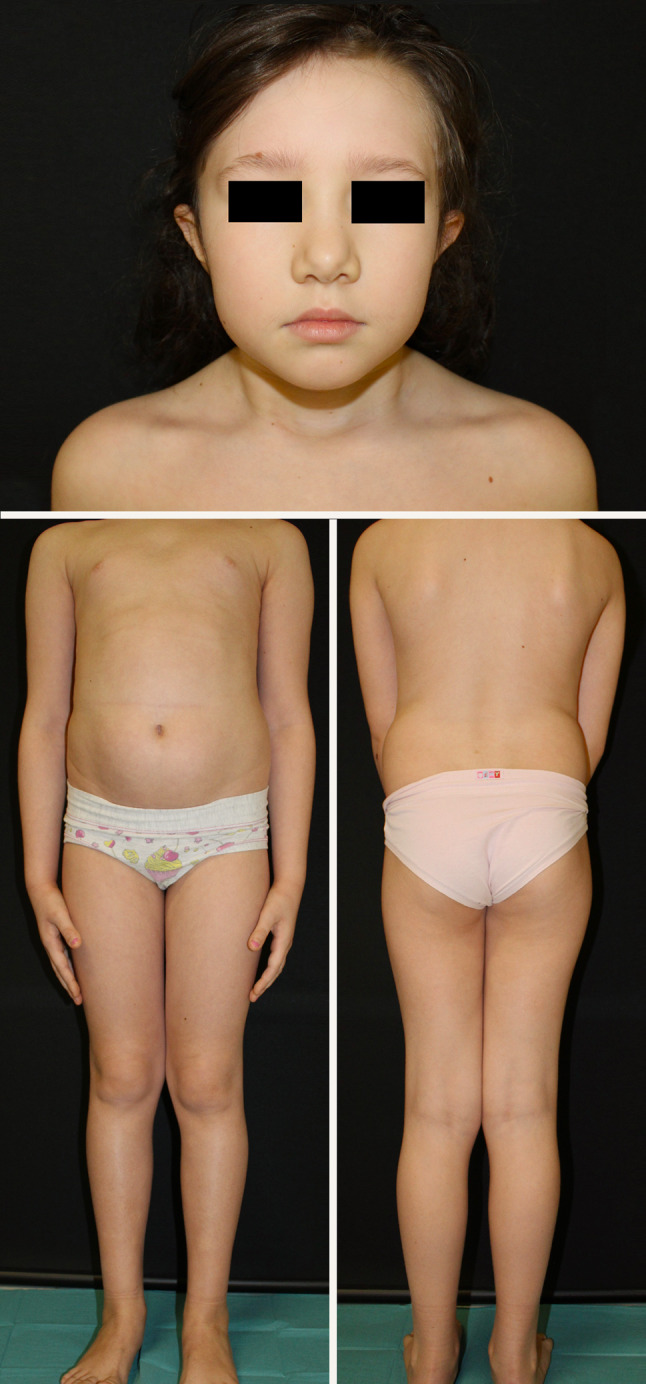
Complete resolution of skin manifestations. Clinical (GPPASI 0, GPPGA 0, NRS pruritus 0, cDLQI 0) and symptomatological remission at week-8 of treatment with ixekizumab

A 7-years-old girl, with history of plaque-psoriasis since the age of 6 months and no comorbidities, presented to our attention for the sudden appearance of erythematous patches surmounted by pustules on her trunk and lower limbs, following repeated episodes of purulent tonsillitis (Fig. 1A). A clinical diagnosis of generalized pustular psoriasis (GPP) was made [1]. GPP Area and Severity Index (GPPASI) [6] at baseline was 13.6, and GPP Physician Global Assessment (GPPGA) was 3^6^. Cyclosporine was started at a dosage of 3.5 mg/kg/day (body weight: 30 kg), with no clinical benefit and progression to a severe sub-erythrodermic state after 2 weeks (GPPASI 25, GPPGA 4) (Fig. 1B-D). The flare was accompanied by intense itching (NRS pruritus 10), pain and strong impact on the patient’s quality of life (Children’s Dermatology Life Quality Index, cDLQI 21). The patient experienced systemic symptoms such as mild hyperpyrexia and malaise. Blood tests showed leukocytes 21.70 × 10^3/uL, neutrophils 16.00 × 10^3/uL and the C-reactive Protein (CRP) value was 2.29 mg/dL. Genetic testing ruled out pathogenic variants in genes involved in the pathogenesis of GPP (*IL36RN*, *CARD14* and *AP1S)*. Since ixekizumab was recently approved for pediatric use in patients with moderate to severe plaque psoriasis [5], we started treatment with 80 mg ixekizumab as induction dose followed by 40 mg every 4 weeks, in combination with prednisone (12.5 mg/day). After 7 days, significant improvements were observed (GPPASI 11.7, GPPGA 2, NRS pruritus 7, cDLQI 10), and the patient experienced resolution of associated systemic symptoms with appreciable variations of blood tests, reduction in neutrophilic leukocytosis (leukocytes 15.60 × 10^3/uL, neutrophils 5.39 × 10^3/uL) and CRP values (< 0.10 mg/dL). The prednisone dosage was then gradually tapered until discontinuation after 15 days. At week-4 GPPASI was 5, GPPGA 1, NRS pruritus 0 and cDLQI 0. At week-8 the patient achieved complete remission of skin manifestations (GPPASI 0, GPPGA 0, NRS pruritus 0, cDLQI 0) and normalization of blood count (Fig. 2). After achieving stable remission, at week 36 we decided to increase the dosing interval to 6 weeks as dose adjustment. The patient is still on ixekizumab therapy, maintaining complete remission during a 52-week follow-up, without safety concerns.

## Discussion and conclusions

Currently there are no universally accepted, evidence-based guidelines for the treatment of GPP [2]. Advances in understanding genetics and pathogenesis have led to the use of different biologics targeting TNF-alpha, IL-17, IL-23, and IL-12/23 inhibitors and some of them have emerged as potential treatments for GPP, although they are not approved, except for Japan, where some biologics are approved [2]. The only treatment FDA/EMA approved for GPP flares in adults is the IL-36 receptor antagonist, spesolimab [7].

Although GPP can occur in children, this condition is very rare, since it typically emerges in adulthood. According to the existing literature the onset age can range from 20 years to 58 years [3]. Confirming data emerged from a recent Italian multicenter observational study including 66 GPP patients, where the average age was 58.1 ± 14.9 years, and the youngest patient was 14 years old [3].

However, there are no currently approved treatments for children in Europe. Recently an evidence-based review reported the use of anti TNF-alpha agents, ustekinumab, anakinra, canakinumab and secukinumab in 57 pediatric GPP patients, demonstrating a good efficacy profile for anti TNF-alpha agents, some safety issues with infliximab and better outcomes in patients treated with secukinumab, an anti-IL-17 agent [8]. To date, there is no report on the use of ixekizumab in pediatric GPP.

A recent Italian Delphi consensus pointed out that the treatment approach is heterogeneous between clinicians, both in the acute and quiescent phases of disease, and no clear pattern of care emerged in the Italian context; a clear need for additional research to guide treatment during both the acute and chronic phases of GPP emerged. Current treatment of flares is divided between the use of non-biological agents, biological agents, or both types of drugs. Among biological drugs, anti-IL-17 drugs were most frequently used by the panelists during a GPP flare, with anti-IL-23 agents being an alternative therapy for some clinicians [9].

We reported the case of a 7-years-old patient with a history of plaque psoriasis who was successfully treated with ixekizumab, achieving complete remission of cutaneous and systemic manifestations after 8 weeks of treatment, maintained until 52 weeks of observation. Disease severity and clinical characteristics of the GPP flare of our patient were consistent with what reported in the literature [3]. IL-17 is a cytokine produced by T-helper 17 cells that plays a key role in the pathogenesis of inflammatory skin diseases. In particular, IL-17 A acts as a potent inducer of neutrophil recruitment [4]. Ixekizumab, an IL-17 A antagonist, demonstrated efficacy in the treatment of GPP in an open-label, phase III study, that included five Japanese GPP patients as a subgroup; results demonstrated that 4/5 patients achieved a PASI-75 response, and 2/5 had complete remission at week-12, which was maintained for the 52-week treatment period [10]. Although there are no standardized guidelines, in the previously mentioned studies the most widely used non-biologic therapy turned out to be systemic steroids, and the most widely used biologic drug ixekizumab, as in our case [3, 9]. It is not possible, however, to establish how much the use of systemic steroids contributes to achieve complete clinical clearance in combination with biological drugs in our case. Further evidence is essential to overcome these limitations. Interestingly, our patient tested negative for genetic variants in the *IL36RN*, *CARD14* and *AP1S* genes. The genetic bases of GPP are still largely unexplored and further research is needed to better understand the pathogenic mechanisms. This report supports the use of ixekizumab as a safe and effective option, both in the short and long-term, in the treatment of GPP, even at pediatric age. Larger studies are needed to confirm this encouraging real-life experience.

## Electronic supplementary material

Below is the link to the electronic supplementary material.

Supplementary Material 1

Supplementary Material 2

## Data Availability

Not applicable.

## Abbreviations

GPP: Generalized pustular psoriasis
IL: Interleukin
GPPGA: GPP Physician Global Assessment
GPPASI: GPP Area and Severity Index
NRS: Numerical Rating Scale
cDLQI: Children’s Dermatology Life Quality Index
CRP: C-reactive Protein
FDA: Food and Drug Administration
EMA: European Medicines Agency
