# Supplementary material for: Ixekizumab as a successful treatment in pediatric generalized pustular psoriasis
Source: Ital J Pediatr. 2025 Feb 11;51:41. doi: 10.1186/s13052-024-01835-2 (PMC11816982; doi:10.1186/s13052-024-01835-2)
Supplement: Supplementary file 1 — Supplementary Material 1 [file 13052_2024_1835_MOESM1_ESM.pdf]

CARE
case report guidelines

CARE Checklist of information to include when writing a case report

(cc) BY-NC-ND

Topic	Item	Checklist item description	Reported on Line
Title	1	The diagnosis or intervention of primary focus followed by the words "case report"	X
Key Words	2	2 to 5 key words that identify diagnoses or interventions in this case report, including "case report"	X
Abstract (no references)	3a	Introduction: What is unique about this case and what does it add to the scientific literature?	X
	3b	Main symptoms and/or important clinical findings	X
	3c	The main diagnoses, therapeutic interventions, and outcomes	X
	3d	Conclusion—What is the main "take-away" lesson(s) from this case?	X
Introduction	4	One or two paragraphs summarizing why this case is unique (**may include references**)	X
Patient Information	5a	De-identified patient specific information.	X
	5b	Primary concerns and symptoms of the patient.	X
	5c	Medical, family, and psycho-social history including relevant genetic information	X
	5d	Relevant past interventions with outcomes	X
Clinical Findings	6	Describe significant physical examination (PE) and important clinical findings.	X
Timeline	7	Historical and current information from this episode of care organized as a timeline	X
Diagnostic Assessment	8a	Diagnostic testing (such as PE, laboratory testing, imaging, surveys).	X
	8b	Diagnostic challenges (such as access to testing, financial, or cultural)	X
	8c	Diagnosis (including other diagnoses considered)	X
	8d	Prognosis (such as staging in oncology) where applicable	X
Therapeutic Intervention	9a	Types of therapeutic intervention (such as pharmacologic, surgical, preventive, self-care)	X
	9b	Administration of therapeutic intervention (such as dosage, strength, duration)	X
	9c	Changes in therapeutic intervention (with rationale)	X
Follow-up and Outcomes	10a	Clinician and patient-assessed outcomes (if available)	X
	10b	Important follow-up diagnostic and other test results	X
	10c	Intervention adherence and tolerability (How was this assessed?)	X
	10d	Adverse and unanticipated events	X
Discussion	11a	A scientific discussion of the strengths AND limitations associated with this case report	X
	11b	Discussion of the relevant medical literature **with references**.	X
	11c	The scientific rationale for any conclusions (including assessment of possible causes)	X
	11d	The primary "take-away" lessons of this case report (without references) in a one paragraph conclusion	X
Patient Perspective	12	The patient should share their perspective in one to two paragraphs on the treatment(s) they received	X
Informed Consent	13	Did the patient give informed consent? Please provide if requested	Yes ☒ No ☐
