# Supplementary material for: Ixekizumab as a successful treatment in pediatric generalized pustular psoriasis
Source: Ital J Pediatr. 2025 Feb 11;51:41. doi: 10.1186/s13052-024-01835-2 (PMC11816982; doi:10.1186/s13052-024-01835-2)
Supplement: Supplementary file 2 — Supplementary Material 2 [file 13052_2024_1835_MOESM2_ESM.docx]

**REGIONE ABRUZZO**

**UNIVERSITÀ DEGLI STUDI DELL'AQUILA**

**ASL 1 –AVEZZANO-SULMONA-L'AQUILA**

**Presidio Ospedaliero “S. Salvatore”**

**U.O.S.D. DERMATOLOGIA GENERALE ED ONCOLOGICA DU**

*Responsabile: Prof.ssa Maria Concetta Fargnoli*

L’Aquila, November 2^nd^, 2024

To the Editorial Board
*Italian Journal of Pediatrics*

Dear Editor,

Please find enclosed the revised version of our manuscript entitled: “Ixekizumab as a successful treatment in pediatric generalized pustular psoriasis” Maria Esposito, Paolo Antonetti, Emanuele Vagnozzi, Andrea De Berardinis, Roberta Bertelli, Francesco Brancati, Maria Concetta Fargnoli, that we would like considered for publication as a Case Report in the Italian Journal of Pediatrics.

Generalized Pustular Psoriasis (GPP) is an autoinflammatory, multisystemic disease, characterized by a diffuse eruption of neutrophilic pustules on an erythematous base, accompanied by systemic symptoms. Globally, the disease is rare, especially in children. If not properly diagnosed and treated, systemic inflammation and multiorgan involvement in GPP can be life-threatening. Although the exact pathogenesis is unclear, it is known that GPP mainly involves the innate immune system, with autoinflammatory processes and neutrophil activation driven by interleukin (IL)-1, TNF-alpha, IL-17A and, above all, IL-36. However, also IL-17 plays a key role in the pathogenesis of inflammatory skin diseases. In particular, IL-17A acts as a potent inducer of neutrophil recruitment. Ixekizumab is a humanized IgG4 monoclonal antibody that selectively binds the cytokine IL-17A and inhibits its interaction with the IL-17 receptor. We report the case of a 7-year-old patient with GPP who was successfully treated with ixekizumab, achieving complete remission of cutaneous and systemic manifestations after 8 weeks of treatment, maintained until 52 weeks of observation. Interestingly genetic testing ruled out pathogenic variants in genes involved in the pathogenesis of GPP.

We would like to thank the reviewers for the time spent to critically review our manuscript and for the comments and suggestions provided. We confirm that neither the manuscript nor any parts of its content are published in another journal. We appreciate your consideration of our manuscript, and we thank you in advance for your attention in this matter.

Sincerely Yours,

Prof. Maria Esposito

Dermatology, Department of Biotechnological and Applied Clinical Sciences

University of L’Aquila

Via Vetoio, Coppito 2, 67100 L’Aquila, Italy

Tel: +39-0862-368519 - Fax: +39-0862-433433 - email: maria.esposito3@univaq.it
